# Papillary necrosis, fluid intake, and sickle cell nephropathy: lessons for the clinical nephrologist

**DOI:** 10.1007/s40620-024-01991-y

**Published:** 2024-07-11

**Authors:** Domenico Cozzo, Silvio Pianca, Valentina Forni Ogna, Stefania D’Arpa, Pietro Ernesto Cippà, Antonio Bellasi

**Affiliations:** 1 Service of Nephrology, Ospedale Regionale di Lugano, Ospedale Civico, Ente Ospedaliero Cantonale, Via Tesserete 46, 6903 Lugano, Switzerland; 2 Service of Nephrology, Ospedale Regionale di Locarno, Ente Ospedaliero Cantonale, Via Ospedale 1, 6600 Locarno, Switzerland; 3 Faculty of Biomedical Sciences, Università della Svizzera italiana, Lugano, Switzerland; 4 Università Degli studi dell’Insubria, Varese, Italy

**Keywords:** Sickle cell nephropathy, Renal papillary necrosis, Fluid intake, Alcohol consumption

## Abstract

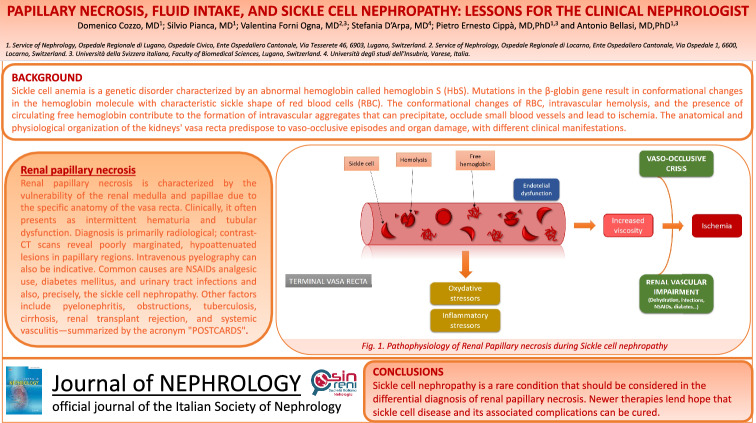

## The case

A 39-year-old patient with a history of anxious depressive syndrome was referred to our renal clinic due to recurring episodes of painless macro-hematuria over the previous month. Approximately 20 days before the consultation, the patient experienced sudden onset hematuria, which persisted for about one week and spontaneously resolved. During the episode of macroscopic hematuria, the patient did not exhibit any symptoms such as colic pain, sore throat, flu-like symptoms, fever, or developed skin lesions.

His past medical history was remarkable for smoking (15-pack-years) and alcohol consumption (about 2 L of beer/day for many years, until about 3–4 months before consultation). He did not take any medication and denies the use of non-steroidal anti-inflammatory drugs (NSAIDs). Since 2018, he had undergone regular annual health check-ups with normal blood and urine tests. His father died of lung cancer, and his mother died of uterine cancer. He had three healthy sisters. His 8-year-old son has asymptomatic hemoglobin S (HbS) heterozygosis.

Clinical examination and blood analysis were unremarkable, renal function was normal for age and sex (creatinine 99 μmol/L, estimated Glomerular Filtration Rate of 86 ml/min, according to the CKD-EPI formula), and he did not present anemia (hemoglobin 139 g/L, mean corpuscular volume of 89 fl). Urinalysis was unremarkable.

Kidney and urinary bladder ultrasounds, cystoscopy, ureteroscopy, and bladder biopsies did not detect any sign of neoplasia. While no kidney stones or evidence of medullary sponge kidneys were observed, contrast-enhanced computed tomography of the abdomen showed images compatible with bilateral papillary necrosis. Hemoglobin electrophoresis showed the presence of HbS with a normal peripheral blood smear, suggesting the patient carries a sickle cell trait.

## Lessons for the clinical nephrologist

This case suggests the occurrence of bilateral papillary necrosis due to a vaso-occlusive crisis in the context of sickle cell nephropathy. Although multiple potential factors such as dehydration, infections, systemic diseases, intense physical activity, or high-altitude flying can trigger the vaso-occlusive crisis, in this case, it is plausible, albeit speculative, that the abrupt cessation of beer consumption (2 L per day) in the 3–4 months before consultation resulted in lower fluid intake leading to a vaso-occlusive crisis. Sickle cell anemia is a genetic autosomal recessive disorder characterized by abnormal hemoglobin called hemoglobin S (HbS). Mutations in the β-globin gene result in conformational changes in the hemoglobin molecule [1]. In homozygous individuals, these changes induce the characteristic sickle shape of red blood cells (RBCs) on peripheral blood smear. Of note, a normal peripheral blood smear does not rule out the disease but suggests a sickle cell trait, as in our case. Clinical and laboratory manifestations vary in individuals with heterozygous (sickle cell trait) and homozygous forms, but both of these presentations can be the cause of systemic disease. The term “Sickle Cell Disease” is used to describe the clinical manifestations that occur as a result of sickle cell anemia [2].

About 300,000 infants worldwide are diagnosed with sickle cell disease every year. The prevalence of the disease is higher in the Mediterranean region, the Middle East, Nigeria, India, and the Democratic Republic of Congo, possibly due to a natural selection process induced by malaria. Indeed, Plasmodium falciparum (malaria pathogen) does not penetrate the sickle-shaped RBCs [3, 4].

In sickle cell disease, the conformational change of RBCs, intravascular hemolysis, and the presence of circulating free hemoglobin contribute to the formation of intravascular aggregates that can precipitate, occlude small blood vessels and lead to ischemia, especially in the presence of endothelial dysfunction, oxidative and inflammatory stressors, and increased blood viscosity. These episodes are also known as vaso-occlusive crises [1, 2].

**Fig. 1:**
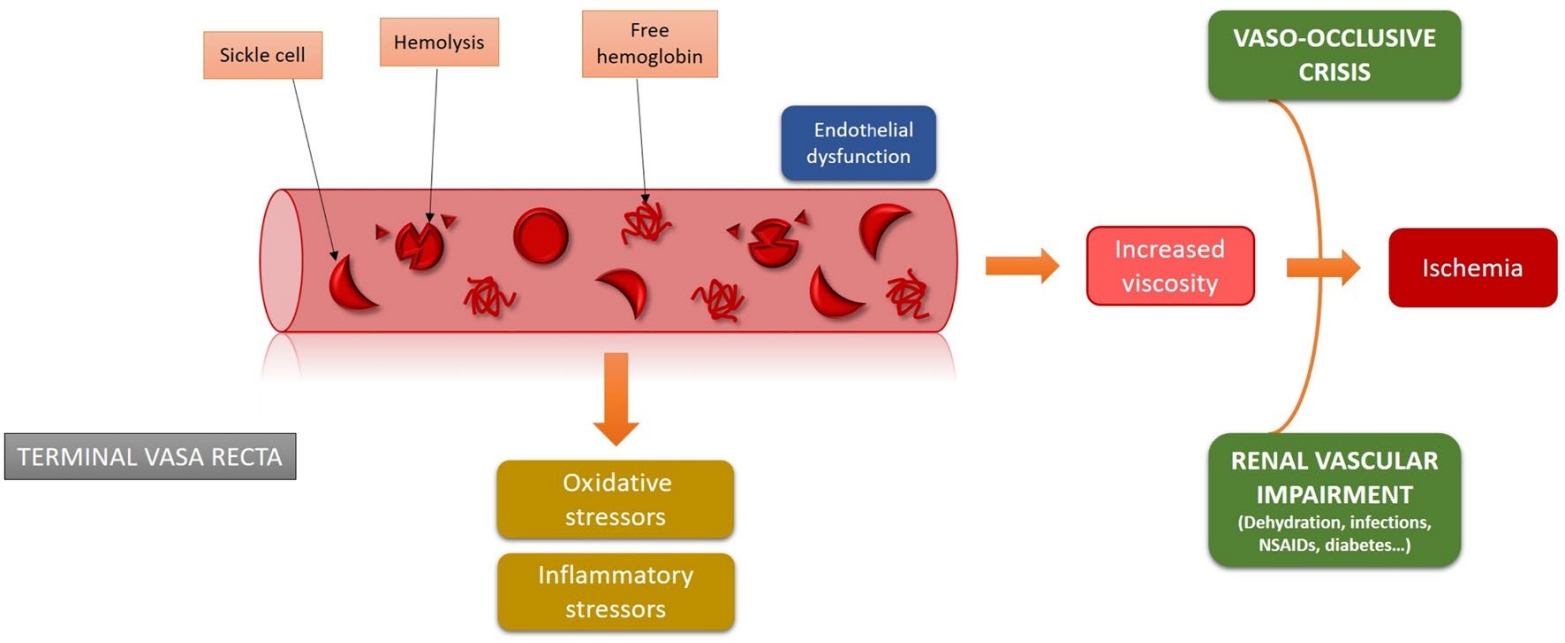
In the sickle cell trait and disease, distal hypoxia promotes hemoglobin polymerization with a conformational change of red blood cells and hemolysis. The presence of free hemoglobin leads to increased oxidative stress with the release of free oxygen radicals. The endothelial dysfunction causes the release of inflammatory and pro-thrombotic molecules. Ischemia further promotes hemoglobin S (HbS) polymerization, worsening distal hypoxia and ischemia and inducing papillary necrosis

Renal manifestations can occur in sickle cell disease and affect quality of life and survival. Indeed, the anatomical and physiological organization of the kidneys' vasa recta and the medullary hypoxic milieu predispose to vaso-occlusive episodes and organ damage (Fig. 1). Sickle cell nephropathy encompasses different clinical manifestations of varying severity due to the involvement of glomerular and tubular disease [4].

Chronic renal ischemia promotes local prostaglandin release, causing arteriolar vasodilation, glomerular hyperfiltration, and albuminuria (detected in about 20% of subjects). All these conditions are associated with a progressive reduction in glomerular filtration rate and chronic kidney disease (CKD) development, as observed in other systemic diseases. About 30% of patients with sickle cell nephropathy eventually develop CKD [2, 7].

Other manifestations of sickle cell nephropathy include impaired urine concentration, acidification capacity and renal potassium excretion secondary to tubular damage [2]. Finally, patients with sickle cell nephropathy appear to be at increased risk of developing renal medullary carcinoma because of chronic cellular damage due to hypoxia and the pro-angiogenic and abnormal anti-apoptotic activity induced by this condition [5].

**Fig. 2:**
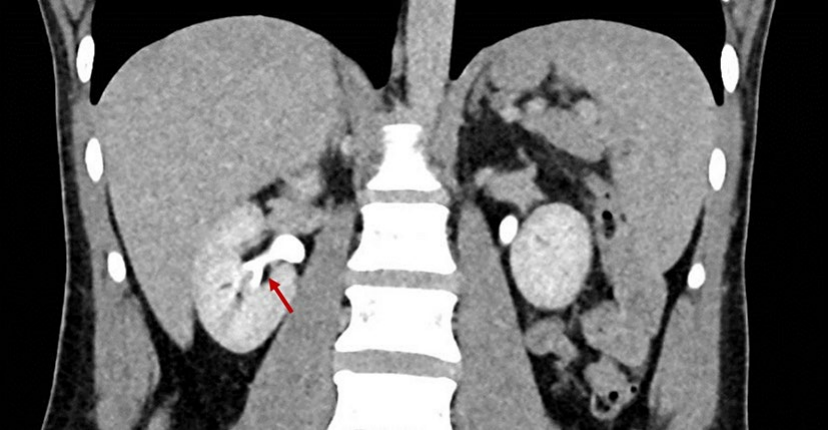
The sagittal view of the contrast-enhanced computed tomography image of the kidney shows the patient's kidney. A typical radiologic sign of renal papillary necrosis is the presence of necrotic cavities filled with contrast, along with the accumulation of contrast around the periphery of the calyces in the papillary region. This is called the "Ball-on-tee" sign (red arrow) [6].

**Fig. 3:**
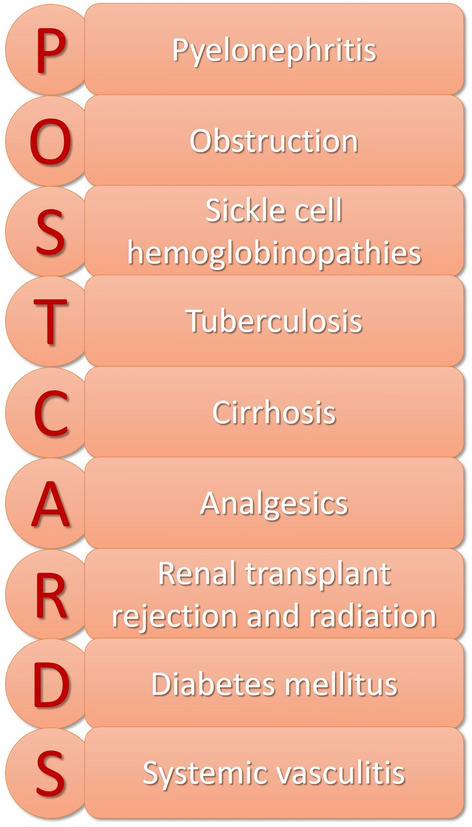
The most frequent etiologies of renal papillary necrosis are summarized by the acronym "POSTCARDS"

Renal papillary necrosis is a rare renal condition characterized by the vulnerability of the renal medulla and papillae due to the specific anatomy of the vasa recta. These small terminal vessels taper towards the renal papilla, leading to limited blood supply and increased risk of ischemia. Clinically, it often presents as intermittent hematuria but can also include hypertension, back pain, and asthenia. Diagnosis is primarily radiological; contrast-enhanced computed tomography scans reveal poorly marginated, hypoattenuated lesions in papillary regions and a "ball-on-tee" sign [6] (Fig. 2). Intravenous pyelography can also be indicative. Common causes are use of NSAIDs, diabetes mellitus, and urinary tract infections as well as the sickle cell nephropathy itself. Other factors include pyelonephritis, obstructions, tuberculosis, cirrhosis, renal transplant rejection, and systemic vasculitis—summarized by the acronym "POSTCARDS" [6, 7] (Fig. 3).

Homozygous patients especially deserve regular monitoring of renal function and proteinuria. The frequency of check-ups depends on clinical manifestations, but should start at a young age. Proteinuria should be monitored closely, since worse renal outcomes have been shown in subjects with sickle cell nephropathy compared to other proteinuric nephropathies with a similar degree of proteinuria [2].

As in other disease conditions, control of traditional and non-traditional cardiovascular risk factors is advisable. Patients with albuminuria can benefit from treatment with Angiotensin-Converting Enzyme (ACE) inhibitors or angiotensin receptor blockers (ARBs). Due to their role in reducing prostaglandins and hyperfiltration, the use of NSAIDs for the treatment of sickle cell nephropathy has been advocated. However, the unfavorable renal impact should be considered [2, 7].

The treatment of sickle cell disease with hydroxyurea may reduce the incidence of vaso-occlusive crises, and reduce proteinuria. Similarly, blood transfusions may have a positive effect on tissue oxygenation. Although the impact on renal outcomes is not established, other treatments such as hematopoietic stem cell transplantation and gene therapies may also preserve the kidney function. In 2023, the US Food and Drug Administration approved two autologous gene therapy products, Lyfgenia® (lovotibeglogene autotemcel by Bluebird Bio) and Casgevy® (exagamglogene autotemcel by Vertex Pharmaceuticals) for the treatment of individuals with sickle cell disease [8].

During vaso-occlusive crises with hematuria, it is important to exclude other clinically relevant causes of hematuria (such as neoplasms). Once the diagnosis of renal papillary necrosis is confirmed, therapy remains supportive and includes intravenous fluids, discontinuation of diuretics, use of analgesics, avoidance of NSAIDs, and blood transfusion if necessary [4, 7].

In summary, sickle cell nephropathy is a rare and elusive condition that should be considered in the differential diagnosis of renal papillary necrosis and renal tubular dysfunction.

## Data Availability

All the data reported in this paper are available upon reasonable request.
